# The Diagnostic Values of Ischemia-Modified Albumin in Patients with Acute Abdominal Pain and Its Role in Differentiating Acute Abdomen

**DOI:** 10.1155/2020/7925975

**Published:** 2020-05-14

**Authors:** Selman Yeniocak, Fatma Saraç, Mustafa Yazıcıoğlu, Nadiye Karabulut, Akın Ünal, Esma Yücetaş, Macit Koldaş, İbrahim Akkoç, Mustafa Ekici, Togay Evrin

**Affiliations:** ^1^University of Health Sciences, Haseki Training and Research Hospital, Emergency Department, Istanbul, Turkey; ^2^University of Health Sciences, Haseki Training and Research Hospital, Clinic of Pediatric Surgery, Istanbul, Turkey; ^3^University of Health Sciences, Bakırköy Sadi Konuk Training and Research Hospital, Emergency Department, Istanbul, Turkey; ^4^University of Health Sciences, Haseki Training and Research Hospital, Family Medicine Department, Istanbul, Turkey; ^5^University of Health Sciences, Haseki Training and Research Hospital, Clinic of Surgery, Istanbul, Turkey; ^6^University of Health Sciences, Haseki Training and Research Hospital, Clinic of Clinical Chemistry, Istanbul, Turkey; ^7^University of Health Sciences, Haseki Training and Research Hospital, Department of Anesthesia and Reanimation, Istanbul, Turkey; ^8^Ufuk University Faculty of Medicine, Department of Emergency Medicine, Ankara, Turkey; ^9^Kilis State Hospital, Emergency Room, Kilis, Turkey

## Abstract

**Aim:**

The aim of this study was to evaluate the diagnostic value of serum ischemia-modified albumin (IMA) levels in patients presenting to the emergency department with acute abdominal pain and its use in differentiating acute surgical abdomen.

**Methods:**

This single-center prospective cross-sectional study included 334 adult patients who presented to the emergency department. These consisted of 194 patients (Group 1) with nontraumatic abdominal pain commencing in the preceding week, who were definitely diagnosed and either hospitalized in a specific department or planned for discharge, and a control group of 140 patients (Group 2).

**Results:**

The mean IMA value of the patients diagnosed with acute appendicitis was statistically significantly higher than that of the control group. The mean IMA value of the patients diagnosed with acute appendicitis, ovarian pathologies, and gastritis-peptic ulcer was statistically significantly higher than that of the nonspecific abdominal pain group.

**Conclusion:**

Serum IMA levels can be used as a diagnostic marker in patients with acute appendicitis. Furthermore, serum IMA levels in patients presenting to the emergency department with abdominal pain may be indicative of patients requiring surgery or of complicated cases, particularly in terms of acute appendicitis and ovarian pathologies.

## 1. Introduction

Acute abdominal pain is a symptom of unknown cause commencing within the previous seven days and indicates a surgical or medical emergency. Since the differential diagnosis of patients presenting with abdominal pain involves numerous potential conditions, it may cause difficulties for the emergency physician [1–3]. Diagnosis can be facilitated by laboratory tests, in addition to history and physical examination. Various markers have been described as capable of use in the differential diagnosis of patients with abdominal pain [4].

Several biochemical markers, including D-dimer, C-reactive protein (CRP), plasma pentraxin-3, and the neutrophil/leukocyte ratio (NLR), have been investigated in terms of the differential diagnosis of patients with acute abdominal pain, particularly in cases requiring surgical intervention. These parameters have been defined as markers of surgery requirements in patients with undiagnosed acute abdominal pain. D-dimer elevation in patients with acute abdominal pain has been reported as a potential marker with high sensitivity for surgical pathologies requiring laparotomy. Another study described CRP levels as a useful parameter for differentiating patients requiring operative or nonoperative treatment among individuals with nonspecific abdominal pain. The plasma neutrophil/leukocyte ratio (NLR) and pentraxin-3 levels have been reported to increase in patients with acute appendicitis and as being of potential use at differential diagnosis [5–8].

The detection in recent years of changes in the structure of serum albumin in ischemic conditions has resulted in the discovery of a novel ischemic marker. The amino N-terminal of albumin is a binding region for transition metals such as cobalt, nickel, and copper [9]. Factors such as hypoxia, acidosis, free radical damage, and membrane impairment that emerge under ischemic conditions reduce the binding of these transition metals to the albumin N-terminal. Albumin subjected to such structural changes is known as ischemia-modified albumin (IMA). Changes in this albumin molecule can be measured colorimetrically with the addition of cobalt to serum. IMA measurement determines the binding capacity of albumin to cobalt and involves the spectrophotometric measurement of cobalt unbound to albumin [10, 11]. IMA was first described in patients with myocardial infarction in 2001 [12]. Although that initial study was performed in patients with myocardial infarction, IMA is not specific to cardiac ischemia. IMA represents 1% to 2% of the total albumin concentration, rising to 8% in patients experiencing ischemia. Some studies have shown that IMA levels increase within minutes after the onset of ischemia, remain elevated for 6 to 12 hours, and return to normal within 24 hours [13]. The high negative predictive value of IMA as an ischemic indicator further enhances its usefulness [14, 15]. Previous studies have shown that IMA levels may rise in cerebral, cardiac, pulmonary, and mesenteric ischemia and in hypoxia cases, as well as in some chronic conditions, such as end-stage renal disease, chronic liver disease, and malignancies, or even in marathon runners with oxidative stress and hypoxic processes. Studies have also shown elevated blood IMA levels due to acute coronary syndrome, pulmonary embolism (PE), mesenteric ischemia, CO intoxication, ischemic stroke, muscle ischemia, pulmonary ischemia, and peripheral vascular diseases. IMA is therefore thought to be capable of use as a diagnostic marker for ischemic and hypoxic processes [16, 17].

The purpose of this study was to evaluate the diagnostic value of serum blood IMA levels in patients presenting to the emergency department with acute abdominal pain and their efficacy in differentiating acute surgical abdomen.

## 2. Materials and Methods

### 2.1. Study Design and Setting

This research was designed as a single-center, prospective, and cross-sectional study. Approval for the study was granted by the Haseki Training and Research Hospital Ethics Committee, Turkey (2016/374). We planned to establish a patient group consisting of individuals aged 18 or over presenting to the emergency department due to nontraumatic acute abdominal pain commencing within the previous week (Group 1) and a control group (Group 2) consisting of a sufficient number of healthy volunteers. All procedures were applied in line with the principles of the Helsinki Declaration. Patients were excluded from the study if they declined to take part or had a condition capable of increasing IMA levels, such as acute coronary syndrome, pulmonary embolism, acute ischemic stroke, mesenteric ischemia, cardiopulmonary resuscitation, peripheral vascular disease, obstructive disease of the main vascular structures, malignancy, hepatic or end-stage renal insufficiency, pregnancy, or major trauma.

### 2.2. Participants

Data related to age, gender, and time of onset of pain before presentation to the emergency department were recorded on patient forms. The blood serum IMA levels of all participants were investigated and recorded as nanogram/milliliter (ng/ml) and were then compared between the patient and control groups. Serum IMA, leukocyte, CRP, and amylase values were compared to determine any correlation between IMA levels and the leukocyte, CRP, and amylase values recorded in the literature for use in acute abdomen patients. Patients with abdominal pain were defined as Group 1 (*n*: 194), and the control group as Group 2 (*n*: 140).

### 2.3. Blood IMA Measurement

Human IMA ELISA kits (Catalog No. CK-E11169, Eastbiopharm, Hangzhou Eastbiopharm Co. Ltd.) were used to determine serum IMA levels, following the manufacturer's instructions. Specimen absorbances were determined on a Biotek ELX800 (Biotek, Winooski, VT, USA) microplate reader at a wavelength of 450 nm. The results were expressed in ng/mL. The minimum detectable level was 2 ng/mL. The costs of the kits used in the study were met by the authors.

### 2.4. Statistical Analysis

Statistical analysis was performed on SPSS 15.0 for Windows software. Descriptive statistics were expressed as number (*n*) and percentage (%) for categorical variables and as mean ± standard deviation (SD), maximum, and minimum values for numerical variables. The Mann–Whitney *U* test was used to compare numerical variables in two independent groups with data showing normal distribution, and the Kruskal–Wallis test in more than two groups. Subgroup analyses in comparisons of more than two groups were performed with the Mann–Whitney *U* test and interpreted with Bonferroni correction. Relationships between numerical variables were investigated using Spearman correlation analysis since parametric test conditions were not established. Numerical variable determining factors were analyzed using regression analysis and the backward method. A value of *p* < 0.05 was regarded as statistically significant.

## 3. Results

**Figure 1:**
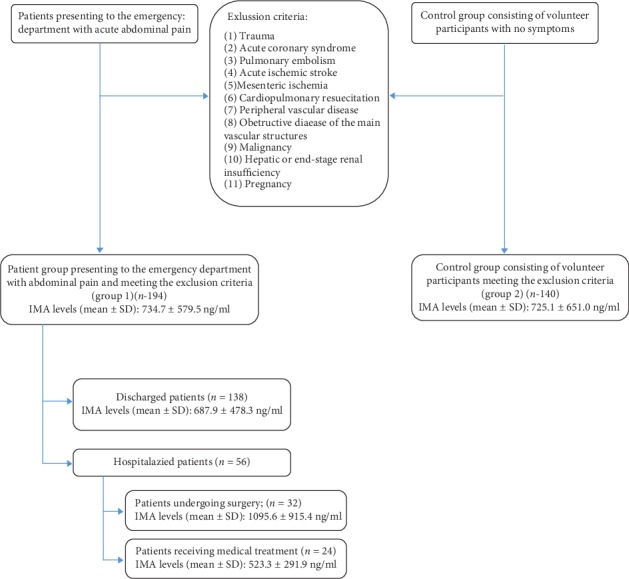
Flowchart of the patient group (Group 1) and control group (Group 2).

Group 1 consisted of 194 patients, 100 (51.5%) men and 94 (48.5%) women with a mean age of 43.0 ± 19.4 years (range, 18–87 years). Group 2 consisted of 140 age- and gender-matched control subjects (Table 1). Mean time from onset of pain to presentation to the emergency department was 21.4 ± 14.9 hours (range, 3–72 hours). The general characteristics and parameter values and a flowchart of the patients in Group 1 are shown in Table 2 and Figure 1.

In Group 1, IMA levels exhibited a significant negative correlation with age (*p*=0.028). No significant correlation was determined in terms of leukocyte, CRP, or amylase values (*p*=0.106, *p*=0.366, and *p*=0.285, respectively).

Mean IMA values were 734.7 ± 579.5 ng/ml (range, 17.3–3192.5 ng/ml) in Group 1 and 725.1 ± 651.0 ng/ml (range, 36.5–3013.2 ng/ml) in Group 2. No statistically significant difference was observed between the groups in respect of mean IMA values (*p* > 0.05). No significant difference was also determined between the genders in terms of mean IMA values (*p* > 0.05) (Table 1).

**Figure 2:**
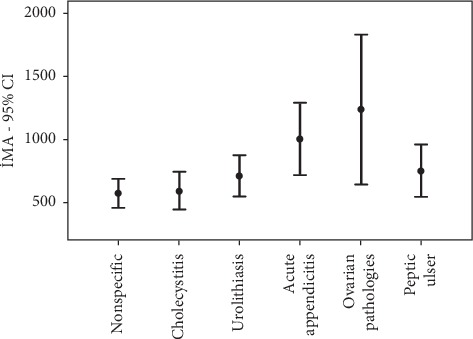
Diagnoses in the emergency department (in order of prevalence) and IMA levels.

The diagnoses of patients in Group 1 included in the analyses were nonspecific abdominal pain, gall bladder diseases, urolithiasis-nephrolithiasis, gastric-peptic ulcer, acute appendicitis (AA), and ovarian diseases. The others could not be included in the analysis (Table 3, Figure 2).

The mean IMA value of patients diagnosed with AA was significantly higher than that of Group 2 (*p* < 0.001) (Table 4). The mean IMA level of patients diagnosed with AA and ovarian diseases (nine cases of ovarian cyst/cyst rupture, one of ovarian torsion, and one of ovarian vein thrombosis) was significantly higher than that of patients diagnosed with nonspecific abdominal pain, and the mean IMA value in patients with AA was significantly higher than that of patients with gall bladder diseases (*p* < 0.001, *p*=0.001, and *p*=0.002, respectively) (Table 5).

One hundred thirty-eight (71.1%) patients were discharged from the emergency department, while 56 (28.9%) were admitted to hospital. The mean IMA value of the discharged patients was 687.9 ± 478.3 ng/ml, which was significantly lower than that of the hospitalized patients (*p*=0.037). The mean IMA value of patients who were hospitalized and received surgical treatment was 1095.6 ± 915.4 ng/ml, which was significantly higher than that of patients receiving medical treatment (*p*=0.014).

**Figure 3:**
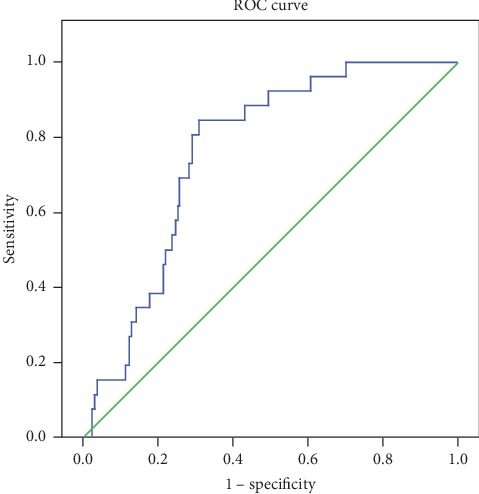
IMA level ROC curve analysis for acute appendicitis and ovarian pathology.

At ROC analysis performed in terms of predictive power for surgery requirements only in AA and ovarian pathologies, the area under the curve was 0.760 (95% CI 0.688–0.832), at a cutoff value of 665.058. For this cutoff value, sensitivity was 80.8%, specificity 70.8%, positive predictive value (PPV) 18.9%, and negative predictive value (NPV) 97.8% (Figure 3).

The mean IMA value in cases of necrotizing AA, as indicated by pathology results, was 1539.9 ± 610.8 ng/ml, significantly higher than the mean IMA value in nonnecrotizing AA cases (735.1 ± 109.0 ng/ml (*p*=0.001)). The mean IMA values of both necrotizing and nonnecrotizing AA cases were both significantly higher than those of the control group (*p*=0.012, *p*=0.004).

## 4. Discussion

No difference was determined in serum IMA levels between the patients presented due to abdominal pain and the control group. However, subgroup analyses revealed significantly higher serum IMA levels in patients diagnosed with AA compared with the control group. Several previous studies support this expected finding [18–22]. This can be explained in terms of ischemia occurring in AA. Occlusion of capillaries and venules and finally venous ischemia develop following increased intraluminal pressure with obstruction of the lumen and associated lumen secretions developing in AA. Arterial ischemia finally occurs, and necrosis and perforation result [23, 24]. IMA levels rise as a result of this ischemia. Elevated serum IMA levels were determined in patients with AA among individuals presenting with abdominal pain in our study.

Serum IMA elevation was also determined in patients with abdominal pain in whom ovarian pathologies were identified. Serum IMA levels of patients with ovarian pathology were significantly higher than those of cases classified as nonspecific abdominal pain. Cases of ovarian pathology included ischemic conditions such as ovarian torsion and cyst rupture. Levels of IMA forming with modification of the N-terminus end in conditions such as free radical damage, energy-related membrane damage, exposure to free iron or copper, acidosis, and hypoxia can increase still further in hypoxic conditions such as ovarian torsion. Several previous studies confirm this. In their experimental study, Karatas Gurgun et al. showed that D-dimer increased more markedly in the early period in animals subjected to ovarian torsion, while IMA increased in the late period. They suggested that IMA is a good biomarker for determining ovarian torsion. Güven et al. determined ovarian torsion in 20 out of 34 patients presenting with pelvic pain and reported high serum IMA levels in all 20. They concluded that serum IMA levels can be useful in determining preoperative ovarian torsion [25–31]. Our finding that IMA levels were significant when ovarian pathology was determined in patients presenting with abdominal pain is in agreement with the previous literature.

On commencing this study, our basic hypothesis was that high and/low serum IMA levels in patients presenting with abdominal pain might serve as a guide to diagnosis and treatment. However, our main finding did not support this. IMA elevation in patients with undiagnosed abdominal pain was not statistically significant. Significant IMA elevation was capable of explaining some causes of abdominal pain, such as AA or ovarian pathology.

However, previous studies of acute appendicitis had already demonstrated that serum IMA elevation can be useful in diagnosis [18–20]. The advantage of our study is that even if other biomarkers are studied in patients presenting with abdominal pain, evaluation together with IMA can assist primary indication of an underlying ischemic pathology. This is because serum IMA elevation will be determined in the presence of existing ovarian torsion or AA in patients presenting with abdominal pain. We therefore think that investigation of serum IMA levels in patients with abdominal pain will be of assistance to the clinician.

Another valuable finding from this study is the higher rate of discharge among patients presenting with abdominal pain and with low IMA levels. Admission and surgery rates were higher among patients with elevated IMA levels. This may be explained in terms of subgroup analyses. We attribute the IMA elevation in AA and ovarian pathologies primarily to ischemia. Serum IMA elevation was also determined in other patient groups such as ileus, aortic aneurysm, acute mesenteric ischemia, and gastrointestinal perforation. The IMA elevation in these patients is again due to ischemia occurring in the gastrointestinal wall. The disadvantage of our study is the low number of patients in this group. However, there have been very few previous studies concerning these patients. In their study of 98 patients, Eroglu et al. determined higher serum IMA levels in patients with aortic pathology compared to a healthy control group. They also suggested that IMA might be a good biomarker for determining aortic pathologies [32–34].

### 4.1. Limitations

The principal limitations of this study are its single-center nature and the low patient number. More definite and extensive results might have been obtained with larger patient numbers. From that perspective, the present research should be regarded as a precursor study and must not be supported by multicenter studies involving wider patient groups.

## 5. Conclusion

In conclusion, serum IMA elevation in patients presenting to the emergency department with abdominal pain can predict ischemic causes of abdominal pain. In addition, based on our study findings, patients with high IMA levels have a greater probability of admission to hospital. Investigation of serum IMA levels in patients presenting to the emergency department with abdominal pain can therefore provide preliminary information concerning progress and severity.

## Figures and Tables

**Table 1:** Patient and control groups, genders, and mean IMA levels.

		Patient group (Group 1)	Control group (Group 2)	*p*
Gender	Male	100 (51.5)	68 (48.6)	0.592
	Female	94 (48.5)	72 (51.4)	

IMA (ng/ml)	(Mean ± SD)	734.7 ± 579.5	725.1 ± 651.0	0.276
	(Min–max)	17.3–3192.5	36.5–3013.2	

min: minimum; max: maximum

**Table 2:** Characteristics and laboratory values of patients in Group 1.

	Patient group (Group 1)
	Mean ± SD (min–max)
Age (years)	43.0 ± 19.4 (15–87)
Duration of pain (hours)	21.4 ± 14.9 (3–72)
Leukocyte (mm^3^)	11403.7 ± 4802.8 (3650–26960)
CRP (mg/L)	45.5 ± 81.7 (0.1–419.1)
Amylase (U/L)	85.4 ± 152.3 (14.6–1509.5)

**Table 3:** Diagnoses in the emergency department and IMA levels.

IMA			
Diagnosis	*n* (%)	Mean ± SD	Median
Group 2	140 (100.0)	725.1 ± 651.0	448.0
Group 1	194 (100.0)	734.7 ± 579.5	521.4
Nonspecific abdominal pain	68 (35.1)	573.5 ± 469.8	403.0
Gall bladder diseases	24 (14.9)	593.3 ± 358.3	502.4
Urolithiasis-nephrolithiasis	29 (12.4)	712.2 ± 435.0	597.4
Gastritis-peptic ulcer	18 (9.3)	750.5 ± 415.8	627.3
Acute appendicitis	15 (7.7)	1003.4 ± 518.1	793.7
Ovarian diseases	11 (5.7)	1235.1 ± 882.6	869.4
The others			
İleus^*∗*^	5 (2.6)	856.1 ± 762.9	652.4
Pancreatitis^*∗*^	5 (2.6)	551.5 ± 232.7	496.0
Hernia^*∗*^	4 (2.1)	445.7 ± 98.8	467.0
Gastrointestinal mass^*∗*^	3 (1,5)	1101.6 ± 1396.5	329.6
Gastrointestinal mass^*∗*^	3 (1.5)	459.6 ± 109.7	464.6
Gastrointestinal perforation^*∗*^	2 (1.0)	761.3 ± 516.5	761.3
Inflammatory bowel disease^*∗*^	2 (1.0)	3088.9 ± 146.6	3088.9
Mesenteric ischemia^*∗*^	1 (0.5)	504.2 ± 762.9	504.2
Mesenteric lymphadenitis^*∗*^	1 (0.5)	1636.2	1636.2
Familial mediterranean fever (FMF)^*∗*^	1 (0.5)	807.6	807.6
Abdominal abscess^*∗*^	1 (0.5)	520.8	520.8
Spleen infarction^*∗*^	1 (0.5)	1828.6	1828.6

^*∗*^Could not be included in the analysis.

**Table 4:** Analysis of the control and patient group diagnoses.

		*p*
	*Group 1*	0.276
*Group 2*	Nonspecific abdominal pain	0.036
	Gall bladder diseases	0.845
	Urolithiasis-nephrolithiasis	0.297
	Gastritis-peptic ulcer	0.038
	Acute appendicitis	<0.001
	Ovarian diseases	0.011

Bonferroni correction *p* < 0.0023.

**Table 5:** Subgroup analysis of the patient group diagnoses.

Subgroup of the patient group diagnoses	Other subgroups of the patient group diagnoses	*p*
Nonspecific abdominal pain	Gall bladder diseases	0.372
	Urolithiasis-nephrolithiasis	0.036
	Gastritis-peptic ulcer	0.006
	Acute appendicitis	**<0.001**
	Ovarian diseases	**0.001**
Gall bladder diseases	Urolithiasis-nephrolithiasis	0.344
	Gastritis-peptic ulcer	0.115
	Acute appendicitis	**0.002**
	Ovarian diseases	0.025
Urolithiasis-nephrolithiasis	Gastritis-peptic ulcer	0.584
	Acute appendicitis	0.028
	Ovarian diseases	0.082
Gastritis-peptic ulcer	Acute appendicitis	0.027
	Ovarian diseases	0.150
Acute appendicitis	Ovarian diseases	0.897

Bonferroni correction *p* < 0.0023.

## Data Availability

The data used to support the findings of this study are included within the article.
